# Detection and annotation of unique regions in mammalian genomes

**DOI:** 10.1093/g3journal/jkae257

**Published:** 2024-11-06

**Authors:** Beatriz Vieira Mourato, Bernhard Haubold

**Affiliations:** Research Group Bioinformatics, Max-Planck-Institute for Evolutionary Biology, August-Thienemann-Str. 2, Plön, Schleswig-Holstein 24306, Germany; Research Group Bioinformatics, Max-Planck-Institute for Evolutionary Biology, August-Thienemann-Str. 2, Plön, Schleswig-Holstein 24306, Germany

**Keywords:** unique regions, transposable elements, enrichment analysis, developmental genes

## Abstract

Long unique genomic regions have been reported to be highly enriched for developmental genes in mice and humans. In this paper, we identify unique genomic regions using an efficient method based on fast string matching. We quantify the resource consumption and accuracy of this method before applying it to the genomes of 18 mammals. We annotate their unique regions (URs) of at least 10 kb and find that they are strongly enriched for developmental genes across the board. We then investigated the subset of URs that lack annotations, which we call “anonymous.” The longest anonymous UR in the Tasmanian devil spanned 83 kb and contained the gene encoding inositol polyphosphate-5-phosphatase A, which is an essential part of intracellular signaling. This discovery of an essential gene in a UR implies that URs might be given priority when annotating mammalian genomes. Our documented pipeline for annotating URs in any mammalian genome is available from the repository github.com/evolbioinf/auger; the additional data for this study are available from the dataverse at doi.org/10.17617/3.4IKQAG.

## Introduction

Transposons were discovered in the mid-1940s by Barbara McClintock, a few years before the discovery of the double-helical structure of DNA by Watson and Crick in 1953. The double helix focused attention on the linear sequence of its nucleotides, while transposons suggest this linearity can be altered. McClintock (1984) observed that the mobilization of transposons often follows unexpected shocks to a cell, for example, through double-strand breaks, infection by retroviruses, or hybridization. McClintock further noted that transposon activation in somatic cells can lead to changes in gene regulation.

When the human genome was first published, its transposon content, roughly 50%, could be quantified with unprecedented precision (International Human Genome Sequencing Consortium 2001). It was noted at the time that the *Hox* clusters had the lowest transposon density of any region of similar size in the genome. The *Hox* clusters are ∼100 kb long and contain about 10 transcriptional regulators that determine an organism’s basic body plan. Mammals have 4 *Hox* clusters, *HoxA*, *HoxB*, *HoxC*, and *HoxD*. The authors of the human genome paper speculated that selection against changes in the regulation of *Hox* genes kept these regions free of transposons.

This observation sparked further interest in the functional content of a newly defined category of DNA sequences, transposon-free regions (TFRs) (Simons *et al.* 2005). Apart from containing no transposons, TFRs also contained no satellite DNA, and no more than 20% of their length was homologous to other parts of the genome. This left ∼1,000 regions of at least 10 kb in the genomes of human and mouse covering a 12 Mb, of which 90% were noncoding. The largest TFR in the human genome was 81 kb long and intersected *HoxA*. In general, TFRs were highly enriched for developmental genes and transcription factors (Simons *et al.* 2005).

There are 2 possible mechanisms for maintaining TFRs. The first is structural, transposons might be prevented from inserting in certain regions because the DNA in those regions is packaged into an inaccessible chromatin structure. The second is evolutionary, if transposon insertions are deleterious, they will be selected against, and hence eliminated from the gene pool. Of these 2 possible mechanisms for maintaining TFRs, exclusion and purifying selection, the selection is generally favored (International Human Genome Sequencing Consortium 2001; Simons *et al.* 2005).

If we accept purifying selection as the main mechanism for maintaining TFRs, then transposon insertion is generally deleterious. From this, it follows that the distribution of transposons in the genomes of extant organisms reflects local sensitivity to transposon insertion. This suggests that mammalian genomes can be read as the result of transposon mutagenesis experiments run over evolutionary time.

A year after the first description of TFRs, a novel chromatin state was discovered, characterized by the co-occurrence of activating H3K4me3 marks and repressing H3K27me3 marks (Bernstein *et al.* 2006). These “bivalent regions” are found in the chromatin of embryonic stem cells where they are associated with developmental genes; they vanish as cells differentiate. Moreover, bivalent regions are located in TFRs.

Bivalency has since attracted a lot of attention as it is associated not only with cell differentiation, but also with its opposite, cell dedifferentiation, better known as cancer. The distribution of transposons, on the other hand, is often less well studied (Platt *et al.* 2016). Moreover, TFRs are not simply the complement of transposons, but so far require for their identification a combination of repeat detection and searches for self-homology.

The best-known tool for repeat detection is RepeatMasker, which takes as input a genome sequence and a set of transposon sequences; it then marks the location of the transposons in the genome. On the scale of mammalian genomes, this amounts to a considerable number of homology searches and hence a large computational effort.

However, the toolset of genomics has changed since the inception of RepeatMasker almost 30 years ago (Smit 1996). For example, fast exact matching using suffix trees or suffix arrays has become more accessible through new algorithms (Abouelhoda *et al.* 2002) and software libraries (Fischer and Kurpicz 2017). This has resulted in read mappers like Bwa (Li and Durbin 2009) and genome aligners like Mummer (Marçais *et al.* 2018).

Fast suffix array construction also underlies the program Macle for quickly detecting TFRs (Pirogov *et al.* 2019). Macle, which stands for *match complexity*, is based on maximal matches, that is, matches that cannot be extended without losing the match. Let $m_{o}$ be the observed number of maximal matches that intersect a sliding window on a genome, and $m_{e}$ its expectation for random sequences, then the *match complexity* is defined as


$$C_{m}=\frac{m_{o}-1}{m_{e}-1},$$

where the subtraction of 1 ensures that $C_{m}$ ranges from 0 to an expectation of 1. Given the null distribution of $C_{m}$ (Pirogov *et al.* 2019), regions where $C_{m}$ is indistinguishable from random can be picked. These regions have no homologs elsewhere in the genome and hence are free of transposons that have recently duplicated. However, the regions detected with Macle are also free of any other types of repeats, while they may conceivably contain a single-copy transposon. Hence we call them *unique regions*, URs.

With a sliding window of 10 kb, the human genome was found to contain 1,243 URs covering 17.3 Mb and 772 URs in the mouse genome covering 10.1 Mb (Pirogov *et al.* 2019). These are similar numbers to those found earlier for TFRs in the human and mouse (Simons *et al.* 2005). Moreover, enrichment of URs for developmental genes was >10-fold and highly significant in both organisms (Pirogov *et al.* 2019).

Here, we extend the analysis of URs to a sample of 18 mammalian genomes from 9 orders covering placentals and marsupials. We identify the URs in these genomes and test for functional enrichment. This requires maps between gene identifiers and Gene Ontology (GO) terms for organisms other than human and mouse, where they are readily available. We present a computational workflow for constructing such maps from scratch for any sequenced mammal. Given these maps, they are used in a Monte Carlo test for enrichment. In agreement with previous studies, we find in all 18 genomes investigated that URs are highly enriched for developmental genes. This establishes a strong link in mammals between a now easily observable property of DNA sequences—uniqueness—and function. In addition, we investigate URs that do not intersect any annotations, *anonymous* URs. The longest anonymous UR in the Tasmanian devil turns out to intersect the gene encoding an essential component of intracellular signaling. If anonymous URs can contain essential genes, the careful annotation of anonymous URs may be a simple but effective step toward completing a given genome annotation.

## Material and methods

### Software

All software repositories mentioned in the following sections are located at github.com/evolbioinf.

### Comparing Macle and RepeatMasker

We compared Macle version 0.1 and RepeatMasker version 4.1.5. Macle runs on a single thread. RepeatMasker was run with 10 threads. Both programs were applied to sequence data containing mutations simulated with the program Mutator, which is part of the Biobox repository. Macle was run with 1 kb windows and RepeatMasker with default parameters. Macle runs on an index of the input file, and the 18 Macle indexes used throughout this study are available from the accompanying dataverse.

### Sequence data and phylogeny

We picked mammalian orders for which we found at least 2 genomes assembled to chromosome level. This resulted in a sample of 9 orders and hence 18 genomes, which are listed in Supplementary Table 1. We used the program Datasets supplied by the NCBI to download these genomes, their GFF3 annotation files, and their proteomes. We describe the details of how to download the data and to extract URs in our repository Auger, for “analyze unique genomic regions.”

We calculated the phylogeny of the 18 genomes sampled from their *HoxD* regions, which were aligned with Mafft (Katoh *et al.* 2005). The alignment was subjected to maximum likelihood phylogeny reconstruction with Iqtree2, which we used to search for the best-fitting model and to bootstrap it 1,000 times in slow mode (Kalyaanamoorthy *et al.* 2017).

### Functional enrichment

Functional enrichment was assessed using the programs Annotate, Ego, and Shuffle from our Gin repository. The program Annotate takes genomic intervals extracted with Merwin (Auger repo) from Macle output and a GFF3 file as input and returns a list of genes whose promoters intersect the intervals. Promoters were defined as 2 kb intervals upstream of the transcription start sites (Robertson *et al.* 2007). Instead of promoters, Annotate can also intersect transcripts. The program Ego was used to count the number of genes per GO term.

The graph of enriched GO terms was drawn using the “chart” link of the QuickGO REST API at www.ebi.ac.uk/QuickGO/api/index.html.

#### Monte Carlo test

We used a Monte Carlo test to assess the significance of the gene counts per GO term. Only GO terms with at least 10 genes were included in the analysis. The program Shuffle (Gin repo) was used to shuffle the unique intervals across the genome of origin. Then we annotated them again and counted the frequency with which a gene count at least equal to the original count was observed. This frequency is the desired *P*-value, the error probability when rejecting the null hypothesis that the observed gene count is due to chance. The test was carried out with 1 million iterations and the resulting *P*-values were Bonferroni-corrected by multiplication with the number of tests, that is, the number of GO terms with at least 10 genes. GO terms with corrected P≤0.01 were considered enriched.

#### Mapping gene IDs to GO terms

The enrichment analysis just described depends on a map between the gene IDs extracted from the GFF3 file and the GO terms that give them functional meaning. This map is provided by the file gene2go supplied by the NCBI: ftp.ncbi.nih.gov/gene/DATA/gene2go.gz.

**Fig. 1.**
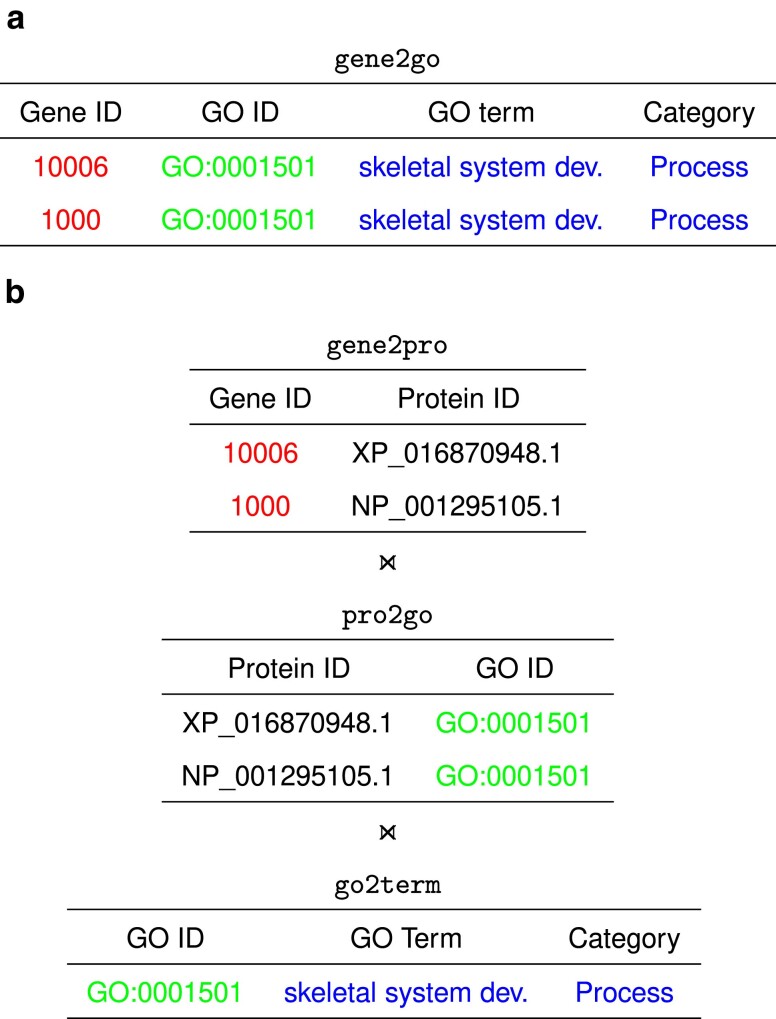
Sample gene2go file a) and its construction b) by joining (⨝) the 3 tables gene2pro, pro2go, and go2term; colors indicate corresponding columns.

The gene2go file originally consists of 8 columns, of which the 4 columns shown in Fig. 1a are used in our analysis. These are Gene ID (red), GO ID (green), GO term (blue), and Category (also blue). Unfortunately, the original gene2go file contains only GO terms for 6 of our 18 mammalian species. So we constructed our own gene2go files. To make results comparable across species, we carried out this construction even for the 6 species represented in the original gene2go file.

As shown in Fig. 1b, the gene2go file is constructed by joining 3 tables. The table gene2pro maps gene IDs to protein IDs and is extracted from the GFF3 file. The table pro2go maps protein IDs to GO IDs and is constructed by subjecting the proteome to homology analysis with the eggNOG software (Cantalapiedra *et al.* 2021). The table go2term maps GO IDs to GO terms and categories and is extracted from the current GO database distributed by the GO consortium via their website geneontology.org; it can be downloaded from https://purl.obolibrary.org/obo/go/go-basic.obo. Like the data handling, the enrichment analysis and the construction of the gene2go files are described in the Auger repo. The 18 gene2go files used in this study are also available from the accompanying dataverse.

## Results

### Comparing Macle and RepeatMasker

We begin by comparing our tool for picking URs, Macle, to an established tool for marking repeats, RepeatMasker. For the purpose of this comparison, we treat picking URs as complementary to picking repeats. We focus on the sensitivity of the respective tools, which we quantify through simulation and application to the human genome.

**Fig. 2.**
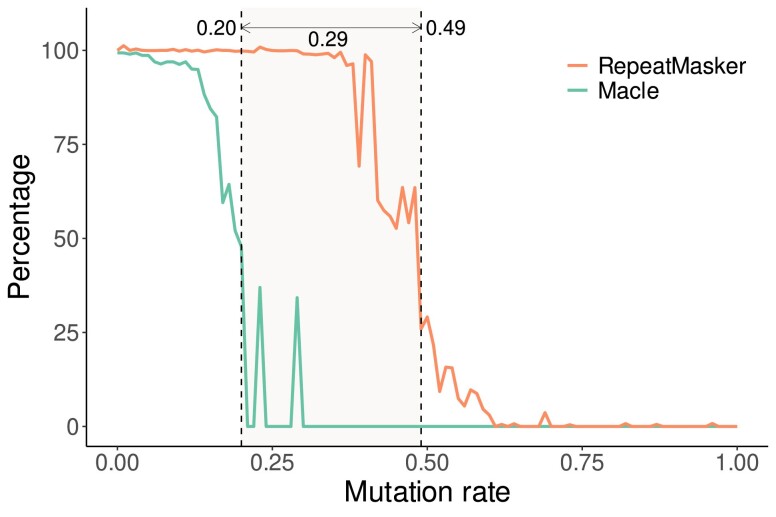
Detecting 2 identical repeats as a function of mutation rate. Shown are the percentage of nucleotides masked by RepeatMasker and the percentage of noncomplex nucleotides calculated by Macle, for a given mutation rate. Vertical lines denote the mutation rate where that percentage first fell below 50%.

For the simulation, we analyze 2 identical copies of the human transposon MER5A1r, which is 3.7 kb long, on the background of a random 25 kb sequence. Figure 2 shows the proportion of the transposon identified by Macle with 1 kb sliding windows and RepeatMasker as a function of the mutation rate. For Macle that proportion declines sharply at 0.2 mutations/site, while for RepeatMasker this happens much later at 0.5 mutations/site.

To compare these simulation results to real data, we applied Macle again with 1 kb windows and RepeatMasker to the human genome. For a start, this revealed the quite different resource requirements of the 2 programs. Macle took 1 h 14 min and 218.0 GB RAM to index the human genome; parsing this index took another 36.0 s and 26.9 GB RAM. In contrast, RepeatMasker required 44 h 19 min and 80.1 GB RAM to mask the human genome, that is, over 30 times longer than the indexing step of Macle but occupying less than half the memory.

**Fig. 3.**
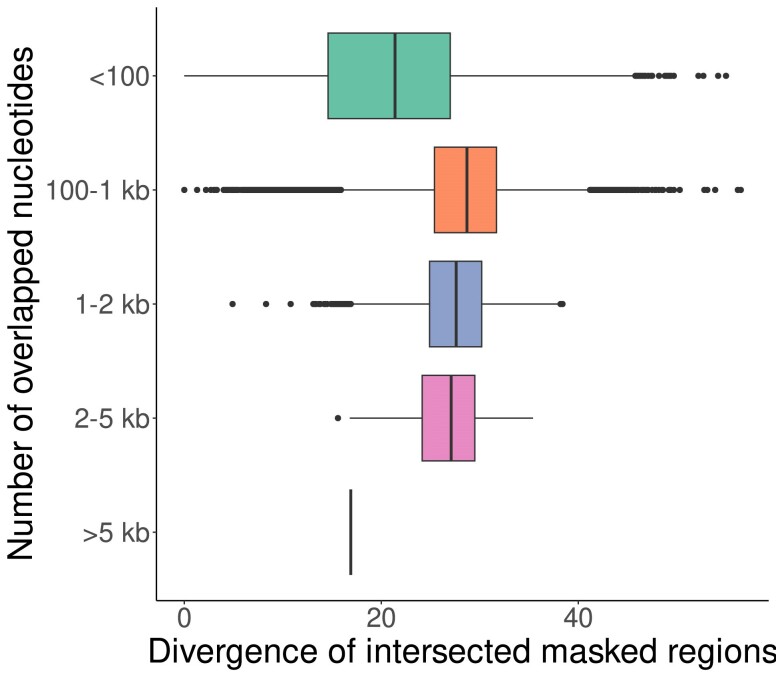
Comparing the divergence rates for overlaps between URs and repeats for overlaps ranging from <100 bp to >5 kb.

Having completed the Macle and RepeatMasker runs on the human genome, we looked up the intersection between masked and URs. From our simulations in Fig. 2, we expected this overlap to be made up of sequences whose closest homolog in the genome has diverged by more than 0.2. Figure 3 shows the divergence of repeats missed by Macle as a function of their divergence rate for 5 categories of repeat lengths. As expected, the divergence of these regions tends to be >0.2 mutations/site, unless they are shorter than 100 bp. The exception to this rule is the single region in the bottom row of Fig. 3. It originates from a single copy of the 7.2 kb human endogenous virus HERV-Fc2 that has diverged by 16.9% from the consensus sequence.

### Unique regions in mammalian genomes

**Fig. 4.**
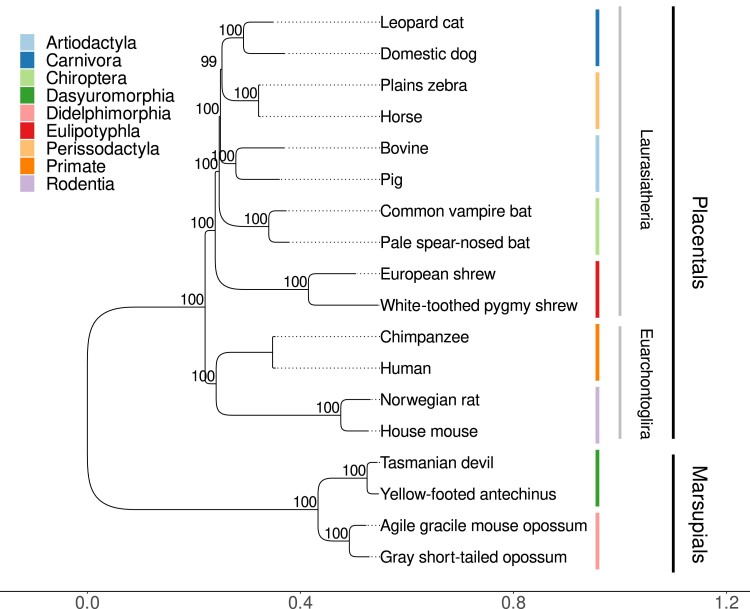
Bootstrapped maximum likelihood tree of the *HoxD* cluster of the 18 species analyzed, 12 placentals and 4 marsupials.

After establishing that the URs identified by Macle are largely free of recent transposon insertions, we proceeded to download the genomes of our 18 target organisms. Figure 4 shows their phylogeny calculated from the *HoxD* cluster. Note the deep split between marsupials and placentals, and the 2 superorders of the Laurasiatheria and the Euarchontoglires.

**Fig. 5.**
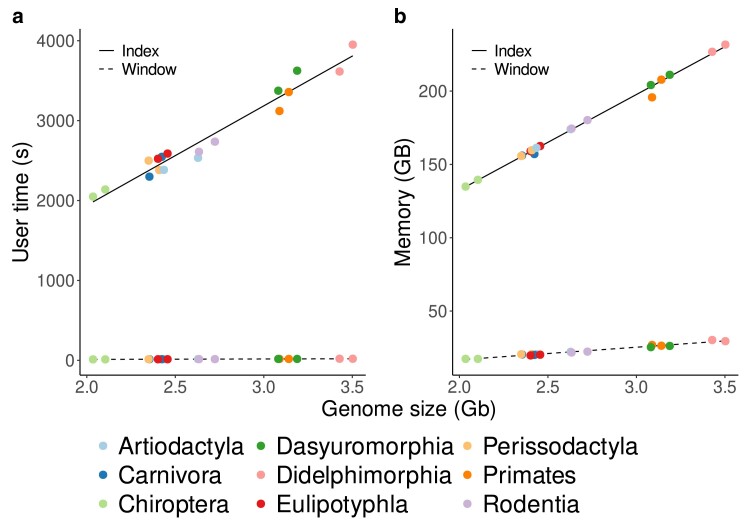
Time a) and memory b) consumption of Macle as a function of genome size when calculating an index (*Index*) or when carrying out a sliding window analysis on an index (*Window*).

In order to pick URs from the downloaded genomes with Macle, they need to be indexed first. Figure 5a shows that the run time of indexing is roughly linear in the genome size, which ranged from 2.1 Gb for the bats (Chiroptera) to 3.7 Gb for the agile gracile mouse opossum (Didelphimorphia), leading to user times between 1,983.3 and 4,056.0 s. Similarly, the memory consumption of indexing is linear in the genome length and ranges from 139.8 to 238.1 GB (Fig. 5b).

Given these indexes, their analysis is much less resource intensive with user times ranging from just 11.8 to 19.8 s and memory requirements from 17.4 to 30.3 GB (Fig. 5). Since using a Macle index is over 170 times faster than computing it, we make the 18 indexes constructed for this study available as part of the accompanying dataverse.

### The effect of window size

**Fig. 6.**
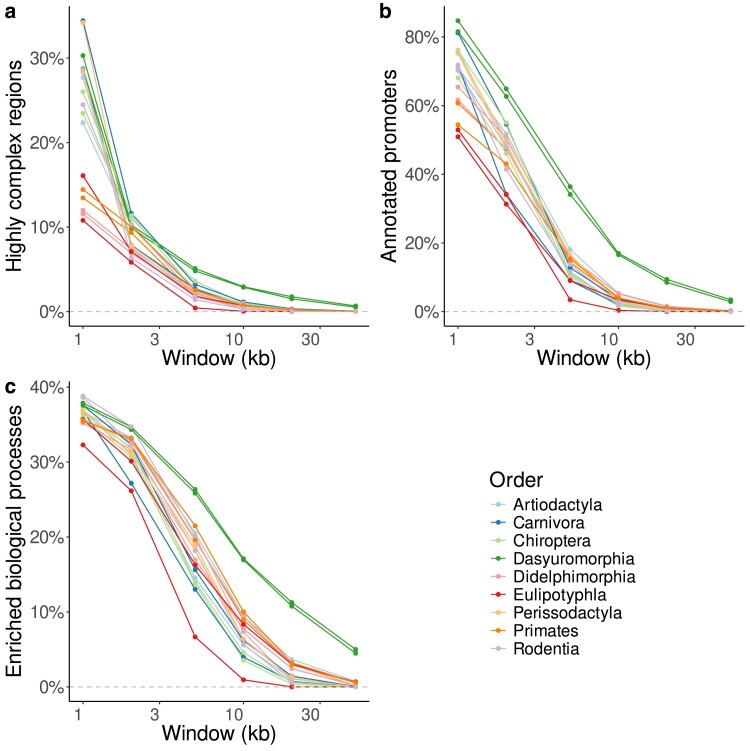
Highly complex, i.e. unique, regions in 18 mammalian genomes as a function of the sliding window size. a) Total length, b) annotated promoters, and c) enriched biological processes.

To explore the effect of window size on our analysis, we varied the window size from 1 to 50 kb. Figure 6a shows that the proportion of URs declines sharply as a function of window length. For 1 kb windows, the fraction of URs ranges from 34.4% for the leopard cat (Carnivora) to 10.8% for the European shrew (Eulipotyphla). Compare this to the 49.1% of the human genome masked by RepeatMasker, which might imply that the complement, 50.9%, is unique. However, according to Macle with a 1 kb sliding window, only 13.5% of the human genome is unique, because Macle recognizes any repeat, not just the repetitive elements collected in a database. As the window size increases, the fraction of URs declines to between 0 in the European shrew and 0.6% in the Tasmanian devil.

Similarly, the fraction of promoters that intersect URs declines with increasing window length. For 1 kb windows, the fraction of intersecting promoters ranges from 84.7% for the Tasmanian devil (Dasyuromorphia) to 50.9% for the white-toothed pigmy shrew (Eulipotyphla) (Fig. 6b). With 10 kb windows, this is down to an average of 4.7%, with a range from 0.4% for the European shrew to 17.0% for the Tasmanian devil.

Likewise, the number of enriched GO terms decreases continuously with increasing window length. As shown in Fig. 6c, for 1 kb windows, the fraction of enriched GO terms varies between 32.3% for the European shrew (Eulipotyphla) and 38.8% for house mouse (Rodentia). For 50 kb windows, this declines to values between 0 in 8 species (pig, domestic dog, bats, European shrew, plains zebra, and rodents) and 5.0% in the Tasmanian devil (Dasyuromorphia).

Doubling the window size to 2 kb slightly decreases the number of enriched GO terms; now 26.2% of terms are enriched in the european shrew, and 34.1% in the house mouse.

**Fig. 7.**
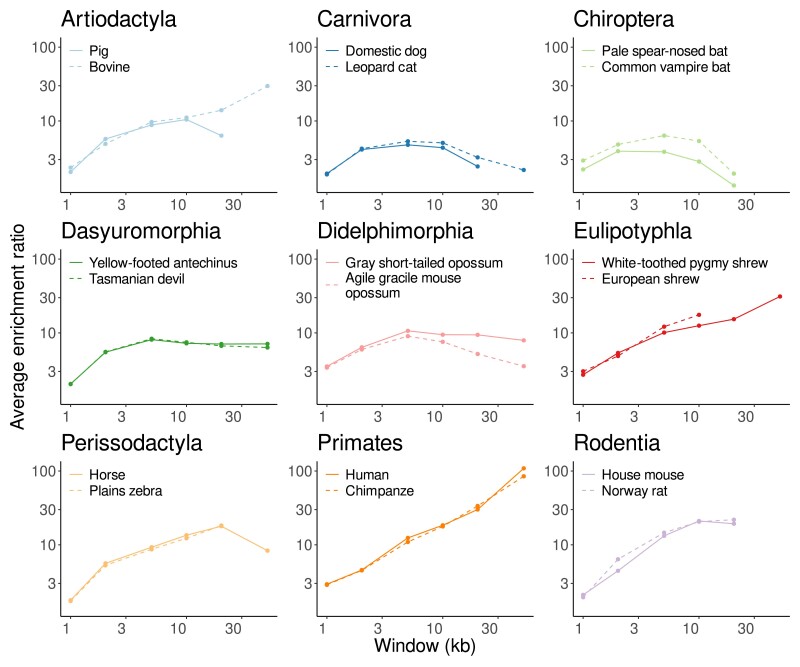
Average enrichment ratio per genome as a function of window size for the 18 mammalian species from 9 genera investigated.

Having surveyed the fraction of enriched GO terms as a function of window length, we now turn to their enrichment ratios. Figure 7 shows the average enrichment ratios as a function of window length for each of the 9 orders. The curves for the 2 species analyzed per order are quite similar. However, the enrichment ratios do not behave uniformly across orders. In almost all of the orders the average enrichment ratio plateaus as a function of window length. In contrast, in the Eulipotyphla and Primates, the average enrichment ratio grows continuously with window size.

### Enrichment of GO terms

The next question we address is, which GO terms are enriched? For this, we restrict our analysis to 10 kb windows, though qualitatively it makes little difference whether we choose 5, 10, or 20 kb as window length (not shown). We concentrated on the GO terms enriched in all 18 species studied, averaged their enrichment ratios across species, and sorted by that average.

**Fig. 8.**
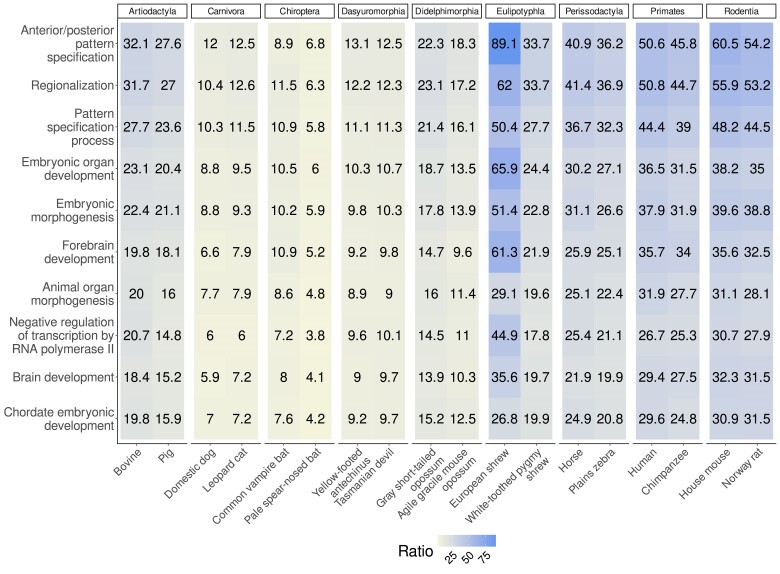
Enriched biological processes shared across the 18 mammalian species investigated. Top 10 processes with the highest average enrichment ratio are shown. The respective enrichment ratio for each species for each term is shown. The darker the hue, the higher the enrichment ratio.

Figure 8 shows the 10 on average most enriched terms, all of which explicitly refer to development, ranging from “Anterior/posterior pattern specification” with an enrichment ratio ranging from 6.8 in the pale spear-nosed bat to 89.1 in the European shrew to “Chordate embryonic development” with an enrichment ratio ranging from 4.2 in the pale spear-nosed bat to 31.5 in the Norwegian rat.

**Fig. 9.**
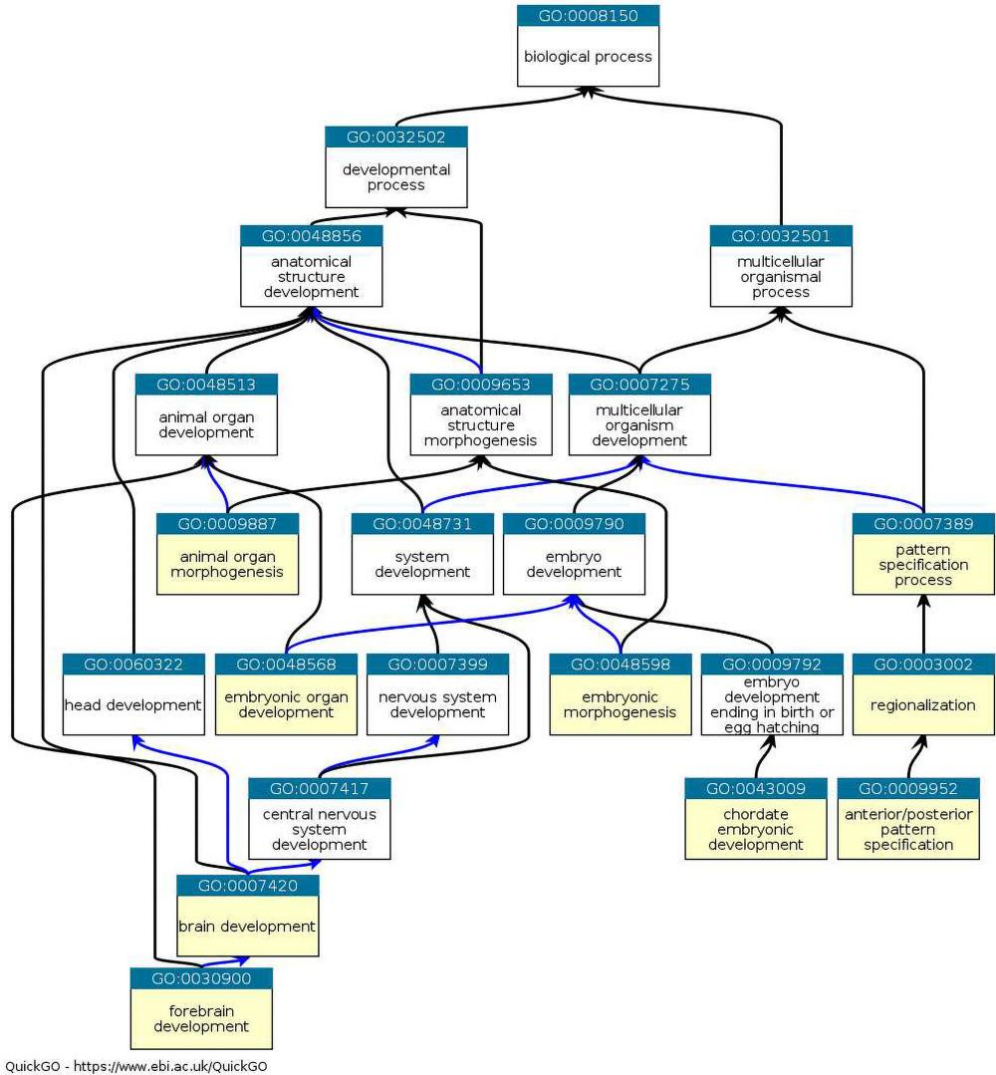
The graph of the 10 most enriched GO terms shown in Fig. 8, except for “Negative regulation of transcription by RNA polymerase II,” for genes with promoters that intersect URs in mammalian genomes; enriched terms are in yellow, black arrows indicate an “is a” relationship, and blue arrows indicate a “part of” relationship.

Figure 9 shows the graph of the 10 most enriched “biological processes” among the GO terms, except for “Negative regulation of transcription by RNA polymerase II,” as its inclusion would have made the graph too large to read. The enriched terms are shown in yellow. Six of them describe a “developmental process.” The remaining 3 form a chain on the right-hand side of the graph and are “multicellular organismal processes” that are part of “multicellular organism development.” The remaining term, “Negative regulation of transcription by RNA polymerase II,” is consistent with the central role of transcriptional regulation in development.

**Fig. 10.**
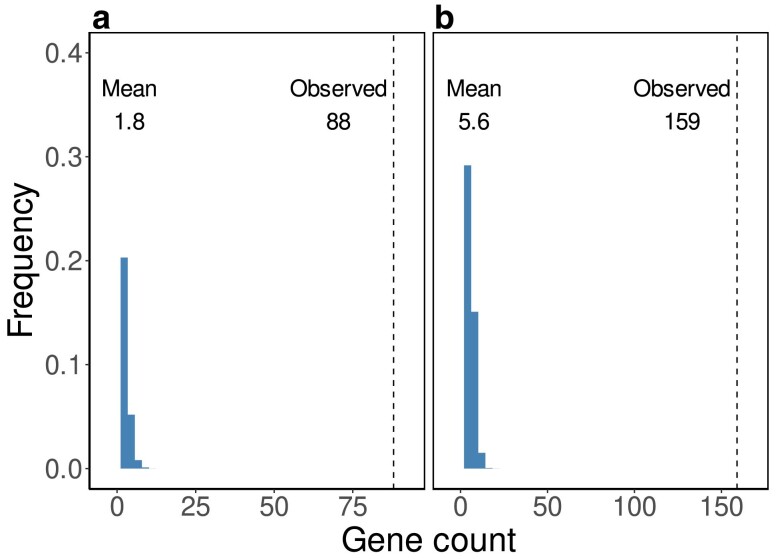
Null distribution of the number of human genes associated with the GO terms “Anterior/posterior pattern specification” a) and “Chordate embryonic development” b), compared with the number of genes observed indicated by the dashed vertical lines.

The Bonferroni-corrected *P*-values of these enrichment ratios were determined by Monte Carlo simulation and are less than the uncorrected $P<10^{-6}$, multiplied by the number of tests, 2,953, yielding P<0.003. This *P*-value is an upper bound that is contingent on the number of iterations in the Monte Carlo test. We used 1 million iterations, a greater number would have reduced the *P*-values. To gain some intuition for the true *P*-values, we plot the null distribution used in the Monte Carlo tests in human for the 2 GO terms “Anterior/posterior pattern specification” and “Chordate embryonic development,” which are the top and bottom terms in Fig. 8. Figure 10a shows the null distribution of the number of genes found for “Anterior/posterior pattern specification” compared with the observed value, 88. Clearly, the null distribution and the observed value are well separated. Similarly, Fig. 10b shows the null distribution of the number of genes found for “Chordate embryonic development,” compared with the observed value, 159. This time the upper bound of the null distribution is even further removed from the observed value, implying an even smaller true *P*-value.

### Anonymous URs

Anonymous URs are those that intersect neither known promoters nor transcripts. In our final analysis, we concentrated on the longest anonymous UR per organism. These ranged from 83 kb in the Tasmanian devil to 10 kb in the European shrew (Supplementary Table 2). To investigate their functional content, we used Blastx to compare them to the SwissProt database. This resulted in 4 hits with $E<10^{-5}$ (not shown). Two of these were against LINE-1 elements, one was to the guanine nucleotide exchange factor in an unplaced contig of the genome of the gray short-tailed opossum. However, the gray short-tailed opossum has a canonical version of the gene encoding this protein on chr6:167,317,046–167,496,814. The most significant hit we found ($E=1.8\times 10^{-13}$) was between the Tasmanian devil UR and human inositol polyphosphate-5-phosphatase A, INPP5A.

INPP5A is 1 of 10 mammalian inositol 5-phosphatases, enzymes that play a crucial role in intracellular signaling (Pirruccello and De Camilli 2012). INPP5A is special among the inositol 5-phosphatases, as it acts on soluble rather than membrane-bound inositol polyphosphates. Of the 18 mammalian genomes we studied, 17 have an annotated INPP5A. Given our Blastx results, the most likely explanation for the apparent absence of INPP5A from the genome of the Tasmanian devil is that its annotation is incomplete in this respect.

## Discussion

URs were of immediate interest at the beginning of the era of mammalian genomics (Simons *et al.* 2005). As noted at the time, URs are enriched for developmental genes and their chromatin is enriched for bivalent markings, and thus silenced but poised for activation (Bernstein *et al.* 2006).

In the years since, RepeatMasker tracks have become a standard feature of genome browsers, and chromatin marks are widely studied as a crucial link between genotype and phenotype. However, URs have not been investigated much since then. The aim of our project was to revive the study of URs by providing simple and efficient tools for their detection and annotation, and by analyzing URs in 18 mammalian genomes (Fig. 4).

As to detecting URs, we used a published tool, Macle, to calculate the match complexity, $C_{m}$, which has an expectation of 1 in DNA regions that cannot be distinguished from random (Pirogov *et al.* 2019). Such regions have no close homologs in the rest of the genome, they are unique.

Since the properties of such regions are not as intuitive as, say, the notion of missing Blast hits, we compared the alignment-based tool RepeatMasker with Macle. Being alignment-free makes Macle much faster than RepeatMasker, but also less sensitive (Fig. 2). As a result, the regions identified are only free of recent transposon insertions, while transposons with a divergence >0.2 escape detection (Fig. 3). Moreover, the $C_{m}$ is only based on repeats, not specifically on a particular type of repeat like transposons, which in the human genome meant that a singleton transposon, HERV-Fc2, was also counted as unique, while RepeatMasker flagged it.

The separation between indexing and searching implemented in Macle is standard in string-based bioinformatics tools like Blast and fast read mappers. In the case of Macle, indexing allows the sliding window analysis to run in seconds, while indexing takes roughly an hour (Fig. 5a). Similarly, the memory consumption of indexing is roughly 2 orders of magnitude greater than that of the sliding window analysis of the index (Fig. 5b).

The sliding window analysis is highly sensitive to window length, where an increase leads to a decrease in yield (Fig. 6a). This corresponds to our intuition that a long region is less likely to escape transposon insertions over long periods of time than a short region. With decreasing yield, the proportion of intersecting promoters also decreases from more than half to 0 (Fig. 6b). Similarly, the proportion of enriched biological processes declines from over 30% to 0 with increasing window length (Fig. 6c).

For each of the 9 mammalian orders we investigated, the 2 genomes sampled closely tracked each other’s graph of the average enrichment ratio as a function of window length (Fig. 7). Given that the longest URs contain loci like the *Hox* clusters, that is, a large number of genes of 1 narrow functional category, we expected a steady increase in enrichment ratio as a function of window length. However, across the orders, the trajectories varied between plateauing in most orders, and steadily increasing in primates and shrews (Eulipotyphla).

Still, the classical observation that URs are enriched for developmental genes (Simons *et al.* 2005) is true for all 18 taxa investigated (Figs 8 and 9). Now, URs are not the only sequence property associated with function, CpG islands are a classical sequence feature associated with transcription initiation in vertebrates (Deaton and Bird 2011). It was previously found that in the human and mouse, and 88% of URs at least 10 kb long intersect a CpG island (Pirogov *et al.* 2019). This large overlap between the 2 features might suggest that URs are essentially CpG islands. However, the specific enrichment for developmental genes observed in URs is only true for CpG islands longer than 2 kb (Elango and Yi 2011). The overlap between URs and long CpG islands is much smaller than between URs and CpG islands in general. In the human, 35% of URs intersect CpG islands of 2 kb or more; in the mouse, this fraction is down to 19% (Pirogov *et al.* 2019).

We called URs without any intersecting promoters or transcripts “anonymous.” As a quick check of their functional content, we compared them to SwissProt, a carefully curated sample of proteins. Our expectation was that we would find nothing, annotation by homology being a standard component of annotation pipelines. Nevertheless, in the 83 kb UR of the Tasmanian devil we found a hit to the signaling enzyme INPP5A. This enzyme is known in the other 17 genomes investigated. We are thus confident that we have located an essential, albeit hitherto missing, gene in the genome of the Tasmanian devil. This specific discovery implies the more general point that annotating anonymous URs, which make up only a small fraction of any mammalian genome (Fig. 6a), might be a simple way to ensure a full complement of the essential genes located in URs.

Our analysis of anonymous URs only scratches the surface of what could be done, including annotation by nucleotide Blast instead of protein Blast, and the identification of transcription factor binding sites (Bailey *et al.* 2015).

To conclude, URs in the genomes of humans and mice were 20 years ago discovered to be enriched for developmental genes (Simons *et al.* 2005). However, the detection of URs has remained difficult due to the large size of mammalian genomes. Here, we have shown that Macle facilitates the detection of URs on various scales and that the workflow implemented in the Augre repository allows their efficient annotation and enrichment analysis. The importance of long URs is underscored by the general observation that they are highly enriched for developmental genes, and by the specific discovery that the longest anonymous UR in the Tasmanian devil contains a hitherto unannotated essential component of intracellular signaling.

## Supplementary Material

jkae257_Supplementary_Data

## Data Availability

All relevant data for this study are available from the dataverse at doi.org/10.17617/3.4IKQAG. Our documented pipeline for finding and annotating unique regions in mammalian genomes is available from the repository github.com/evolbioinf/auger. Supplemental material available at G3 online.

## References

[jkae257-B1] Abouelhoda M, Kurtz S, Ohlebusch E. 2002. The enhanced suffix array and its applications to genome analysis. In: Guigó, R., Gusfield, D, editors. Lecture Notes in Computer Science. Vol. 2452. Berlin, Heidelberg: Springer-Verlag. p. 449–463. 10.1186/1471-2105-9-476 PMC3224568 19014477

[jkae257-B2] Bailey TL, Johnson J, Grant CE, Noble WS. 2015. The MEME suite. Nucleic Acids Res. 43(W1):W39–W49. doi:10.1093/nar/gkv416 25953851 PMC4489269

[jkae257-B3] Bernstein BE, Mikkelsen TS, Xie X, Kamal M, Huebert DJ, Cuff J, Fry B, Meissner A, Wernig M, Plath K, et al 2006. A bivalent chromatin structure marks key developmental genes in embryonic stem cells. Cell. 125(2):315–326. doi:10.1016/j.cell.2006.02.041 16630819

[jkae257-B4] Cantalapiedra CP, Hernandez-Plaza A, Letunic I, Bork P, Huerta-Cepas J. 2021. eggNOG-mapper v2: functional annotation, orthology assignments, and domain prediction at the metagenomic scale. Mol Biol Evol. 38:5825–5829. doi:10.1093/molbev/msab293 34597405 PMC8662613

[jkae257-B5] Deaton AM, Bird A. 2011. CpG islands and the regulation of transcription. Genes Dev. 25:1010–1022. doi:10.1101/gad.2037511 21576262 PMC3093116

[jkae257-B6] Elango N, Yi SV. 2011. Functional relevance of CpG island length for regulation of gene expression. Genetics. 187:1077–1083. doi:10.1534/genetics.110.126094 21288871 PMC3070517

[jkae257-B7] Fischer J, Kurpicz F. 2017. Dismantling DivSufSort. In: Holub J, Žd’árek J, editors. Proceedings of the Prague Stringology Conference 2017. p. 62–76. https://www.stringology.org/event/2017/p07.html

[jkae257-B8] International Human Genome Sequencing Consortium . 2001. Initial sequencing and analysis of the human genome. Nature. 409:860–921. doi:10.1038/35057062 11237011

[jkae257-B9] Kalyaanamoorthy S, Minh BQ, Wong TKF, von Haeseler A, Jermiin LS. 2017. ModelFinder: fast model selection for accurate phylogenetic estimates. Nat Methods. 14(6):587–589. doi:10.1038/nmeth.4285 28481363 PMC5453245

[jkae257-B10] Katoh K, Kuma K, Toh H, Miyata T. 2005. MAFFT version 5: improvement in accuracy of multiple sequence alignment. Nucleic Acids Res. 33(2):511–518. doi:10.1093/nar/gki198 15661851 PMC548345

[jkae257-B11] Li H, Durbin R. 2009. Fast and accurate short read alignment with Burrows–Wheeler transform. Bioinformatics. 25:1754–1760. doi:10.1093/bioinformatics/btp324 19451168 PMC2705234

[jkae257-B12] Marçais G, Delcher AL, Phillippy AM, Coston R, Salzberg SL, Zimin A. 2018. MUMmer4: a fast and versatile genome alignment system. PLoS Comput Biol. 14:e1005944. doi:10.1371/journal.pcbi.1005944 29373581 PMC5802927

[jkae257-B13] McClintock B . 1984. The significance of responses of the genome to challenge. Science. 226:792–801. doi:10.1126/science.15739260 15739260

[jkae257-B14] Pirogov A, Pfaffelhuber P, Börsch-Haubold AG, Haubold B. 2019. High-complexity regions in mammalian genomes are enriched for developmental genes. Bioinformatics. 35(11):1813–1819. doi:10.1093/bioinformatics/bty922 30395202 PMC6546125

[jkae257-B15] Pirruccello M, De Camilli P. 2012. Inositol 5-phosphatases: insights from the Lowe syndrome protein OCRL. Trends Biochem Sci. 37(4):134–143. doi:10.1016/j.tibs.2012.01.002 22381590 PMC3323734

[jkae257-B16] Platt RN, II, Blanco-Berdugo L, Ray DA. 2016. Accurate transposable element annotation is vital when analyzing new genome assemblies. Genome Biol Evol. 8:403–410. doi:10.1093/gbe/evw009 26802115 PMC4779615

[jkae257-B17] Robertson G, Hirst M, Bainbridge M, Bilenky M, Zhao Y, Zeng T, Euskirchen G, Bernier B, Varhol R, Delaney A, et al 2007. Genome-wide profiles of STAT1 DNA association using chromatin immunoprecipitation and massively parallel sequencing. Nat Methods. 4:651–657. doi:10.1038/nmeth1068 17558387

[jkae257-B18] Simons C, Pheasant M, Makunin IV, Mattick JS. 2005. Transposon-free regions in mammalian genomes. Genome Res. 16:164–172. doi:10.1101/gr.4624306 16365385 PMC1361711

[jkae257-B19] Smit AFA . 1996. The origin of interspersed repeats in the human genome. Curr Opin Genet Dev. 6(6):743–748. doi:10.1016/S0959-437X(96)80030-X 8994846

