# Supplementary material for: Detection and annotation of unique regions in mammalian genomes
Source: G3 (Bethesda). 2024 Nov 6;15(1):jkae257. doi: 10.1093/g3journal/jkae257 (PMC11708210; doi:10.1093/g3journal/jkae257)
Supplement: jkae257_Supplementary_Data [file jkae257_supplementary_data.pdf]

Supplementary Material for *Detection and Annotation of Unique Regions in Mammalian Genomes*

Beatriz Vieira Mourato, and Bernhard Haubold

Research Group Bioinformatics, Max-Planck-Institute for Evolutionary Biology, Plön, Germany

November 1, 2024

Table S1: Genome accessions, corresponding mammalian order for each species, total genome size.

#	Accession	Order	Species	Common name	Size (Gb)
1	GCF_002263795.2	Artiodactyla	*Bos taurus*	Bovine	2.7
2	GCF_000003025.6	Artiodactyla	*Sus scrofa*	Pig	2.5
3	GCF_011100685.1	Carnivora	*Canis lupus familiaris*	Domestic dog	2.5
4	GCF_016509475.1	Carnivora	*Prionailurus bengalensis*	Leopard cat	2.4
5	GCF_004126475.2	Chiroptera	*Phyllostomus discolor*	Pale spear-nosed bat	2.1
6	GCF_022682495.1	Chiroptera	*Desmodus rotundus*	Common vampire bat	2.1
7	GCF_016432865.1	Dasyuromorphia	*Antechinus flavipes*	Yellow-footed antechinus	3.2
8	GCF_902635505.1	Dasyuromorphia	*Sarcophilus harrisii*	Tasmanian devil	3.1
9	GCF_000002295.2	Didelphimorphia	*Monodelphis domestica*	Gray short-tailed opossum	3.6
10	GCF_016433145.1	Didelphimorphia	*Gracilinanus agilis*	Agile gracile mouse opossum	3.7
11	GCF_024139225.1	Eulipotyphla	*Suncus etruscus*	White-toothed pygmy shrew	2.5
12	GCF_027595985.1	Eulipotyphla	*Sorex araneus*	European shrew	2.4
13	GCF_002863925.1	Perissodactyla	*Equus caballus*	Horse	2.5
14	GCF_021613505.1	Perissodactyla	*Equus quagga*	Plains zebra	2.5
15	GCF_000001405.40	Primates	*Homo sapiens*	Human	3.3
16	GCF_028858775.1	Primates	*Pan troglodytes*	Chimpanzee	3.2
17	GCF_000001635.27	Rodentia	*Mus musculus*	House mouse	2.7
18	GCF_015227675.2	Rodentia	*Rattus norvegicus*	Norwegian rat	2.6

Table S2: The longest anonymous region in each organism investigated; chromosome *u* is *unplaced*.

Assembly	Name	Chromosome	Start	End	Length (kb)
GCF_902635505.1	Tasmanian devil	NC_045427.1 (2)	660,845,372	660,928,371	83
GCF_016433145.1	Agile gracile mouse opossum	NC_058136.1 (X)	61,932,012	61,993,011	61
GCF_022682495.1	Common vampire bat	NC_071393.1 (7)	1,735,042	1,795,041	60
GCF_016432865.1	Yellow-footed antechinus	NC_067404.1 (X)	72,884,124	72,938,123	54
GCF_004126475.2	Pale spear-nosed bat	NC_040917.2 (15)	25,189,025	25,238,024	49
GCF_016509475.1	Leopard cat	NC_057358.1 (B4)	140,638,285	140,675,284	37
GCF_000002295.2	Gray short-tailed opossum	NW_001583633.1 (u)	31,561	65,560	34
GCF_011100685.1	Domestic dog	NC_049238.1 (17)	1,452,570	1,482,569	30
GCF_000003025.6	Pig	NC_010453.5 (11)	77,807,181	77,837,180	30
GCF_028858775.1	Chimpanzee	NC_072412.1 (14)	92,813,178	92,840,177	27
GCF_021613505.1	Plains zebra	NC_060274.1 (8)	129,719,911	129,745,910	26
GCF_002263795.2	Bovine	NW_020192290.1 (u)	169,985	193,984	24
GCF_024139225.1	White-toothed pygmy shrew	NC_064855.1 (8)	123,975,861	123,996,860	21
GCF_000001405.40	Human	NC_000004.12 (4)	10,214,304	10,235,303	21
GCF_002863925.1	Horse	NC_009175.3 (X)	22,668,551	22,686,550	18
GCF_000001635.27	House mouse	NC_000086.8 (X)	56,958,927	56,971,926	13
GCF_015227675.2	Norwegian rat	NC_051339.1 (4)	123,771,487	123,783,486	12
GCF_027595985.1	European shrew	NC_073302.1 (1)	164,574,001	164,584	10
